# Classification of Large-Scale Remote Sensing Images for Automatic Identification of Health Hazards: Smoke Detection Using an Autologistic Regression Classifier

**DOI:** 10.1007/s12561-016-9185-5

**Published:** 2016-11-28

**Authors:** Mark A. Wolters, C. B. Dean

**Affiliations:** 1 Shanghai Center for Mathematical Sciences, Fudan University, 22nd Floor, Guanghua Tower East, 220 Handan Road, Shanghai, China 20043; 2 Department of Statistical and Actuarial Sciences, Western University, Western Science Centre, Room 262, 1151 Richmond Street, London, Ontario, Canada N6A 5B7

**Keywords:** Machine learning, Hyperspectral images, Image segmentation, Autologistic regression, Forest fire smoke

## Abstract

Remote sensing images from Earth-orbiting satellites are a potentially rich data source for monitoring and cataloguing atmospheric health hazards that cover large geographic regions. A method is proposed for classifying such images into hazard and nonhazard regions using the autologistic regression model, which may be viewed as a spatial extension of logistic regression. The method includes a novel and simple approach to parameter estimation that makes it well suited to handling the large and high-dimensional datasets arising from satellite-borne instruments. The methodology is demonstrated on both simulated images and a real application to the identification of forest fire smoke.

## Introduction

In the current Big Data era, one may (naively) be inclined to believe that relevant data are copious and cheaply available regardless of the circumstances. In such a moment, we may remind ourselves that obtaining data of high quality—with sufficient accuracy and precision, and measured representatively over the population in question—is not always easy. One situation of this type is the assessment of exposure to airborne environmental health hazards. In this case, we are interested in the estimation of a spatial field that may cover a large geographic area. The field may be viewed as a two-dimensional region if only surface concentrations are of interest, or as a three-dimensional space if elevation is included.

Direct measurement of the hazard over the entire region is a practical impossibility. The next best thing is to take direct measurements at a set of monitoring stations, and use these point estimates to estimate the rest of the spatial field. The number and location of monitoring stations are typically subject to physical and economic constraints, however, making optimal allocation of resources in this setting an important research topic in its own right (see, e.g. [15]).

While a network of monitoring stations may indeed generate enough information to warrant classification as “big data”, the sparsity of the network in many areas is a limiting factor. Exposure assessment is no longer a question of measurement only, but of prediction as well. Given the monitoring stations’ output, prediction over the spatial field may be done using statistical methods, using suitable computational models of the physical system, or using these two methods in combination [8].

The present work considers how remote sensing imagery from Earth-orbiting satellites might be used as an alternative data source to help study exposure to airborne health hazards. A variety of instruments orbiting the earth are continuously collecting radiometric data that, with appropriate analysis, may provide a useful independent data source. While information from remote sensing instruments might not be as accurate or detailed as that from a monitoring station, the spatial coverage of the imagery is far greater, potentially covering the entire area of interest.

A longstanding example of the use of satellite data to study the atmosphere is the measurement of aerosol optical depth (AOD; see, e.g. [17, 20]). While aerosols are not readily visible to the eye (for example, in colour images of the surface), the estimation of AOD from radiometric data is relatively well established from an understanding of the relevant physical principles. Our methods, by contrast, are designed for situations where a human photointerpreter is able to identify the hazard of interest, but the fundamental understanding is insufficient to permit automated identification of the hazard from first principles. We rely on machine learning techniques applied to labelled samples to develop classifiers that can recognize the hazard in new images.

The nature of remote sensing imagery makes it ripe for application of machine learning techniques. The reasons for this are threefold.

**Fig. 1:**
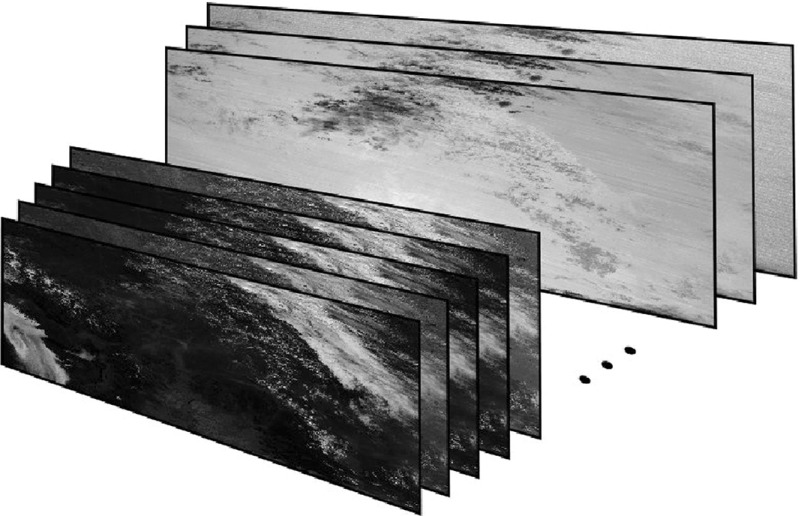
An illustration of a hyperspectral image

First, the data are high dimensional. Images are not only of high resolution (having high pixel counts), but in many cases are *hyperspectral*, consisting of many image planes. As illustrated in Fig. 1, a hyperspectral image with *k* planes is an array stacking *k* greyscale images on top of each other. Each greyscale image represents the scene as measured in a certain wavelength. Hyperspectral images cannot be visualized as a single colour image without loss of information, making them difficult for a human to interpret.

Second, the data are of high throughput. The MODIS data we use for our application in Sect. 5, for example, provide almost whole-globe coverage at 1 km$^2$ per pixel resolution on a daily basis.

Third, the most common task in photointerpretation is segmentation—assigning labels to pixels to divide the image into meaningful categories. But this is just classification, the dominant task in machine learning.

The volume and throughput of data make a complete analysis of the images through the judgement of human experts impractical. At the same time, the task being carried out (classification) is well suited to machine automation. So it makes sense to use a limited set of expert-labelled training images to develop automated routines; if this can be done successfully, complete cataloguing of the remote sensing data as it arrives becomes a reasonable goal.

The application of classification methods to segmentation of hyperspectral images is of course a very broad research goal. To make the present scope more modest, we consider a specific model: the autologistic regression classifier. Further, we develop our methods with a specific application—the identification of smoke from forest fires—in mind. This application, our dataset, previous work, and details of the model are all described in Sect. 2.

Autologistic regression is attractive as a segmentation method because it incorporates spatial associations in a principled, model-based manner; but practical application of the model to large image sets involves daunting computational challenges. Section 3 describes a novel analysis procedure that overcomes these challenges and allows large sets of high-resolution, hyperspectral images to be processed in a reasonable time. The procedure is described in a general context and should be applicable to other problems in remote sensing image processing.

Following the description of the modelling process, Sect. 4 provides experimental evidence of the method’s validity using a collection of simulated images. Section 5 shows how the methods performed on the smoke identification dataset. The paper concludes with two short sections giving discussion and conclusions.

## Background

Below, we first describe the application of interest in more detail, along with the data we have collected. We then summarize previous work that has been done with these data, and describe the autologistic regression model as a natural extension of that work.

**Fig. 2:**
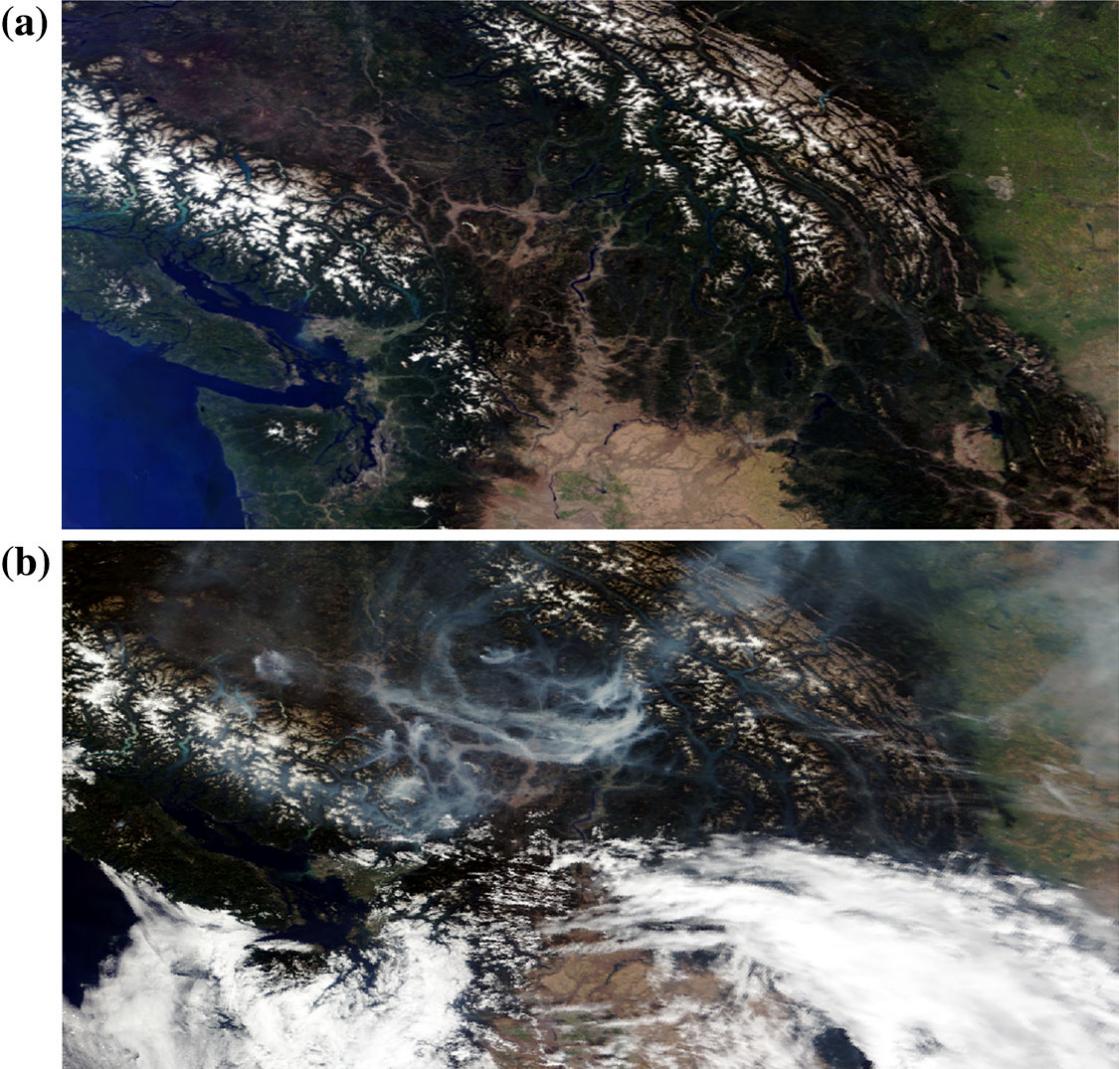
The region of interest, showing **a** a clear-sky composite image, and **b** a typical image during a significant smoke event

### Smoke Identification

Forest fire events can produce great quantities of smoke that can subsequently cover very large geographic areas. Figure 2a shows the region of interest (ROI) for our application. It is a true-colour image constructed from the hyperspectral data (following the guidance in Gumley et al. [9]). This particular image is actually a background image composed by combining the clear-sky portions of 17 individual images and taking the median values of each spectral component at each pixel. Contrast this with Fig. 2b, which shows the same region during a significant smoke event.

The dataset has been described in detail by Wolters and Dean ([27, Sect. 1.2]), so only a summary will be given here. The ROI is centred at Kelowna, Canada, and covers an area of over $8\times 10^5$ km$^2$. There are 143 images covering the fire seasons on consecutive days over three years, and all of the images are 779 pixels high and 1559 pixels wide. The data were collected by the moderate resolution infrared spectroradiometer (MODIS) instrument aboard NASA’s Terra satellite, and consist of $k=35$ spectral bands.

The conventional approach to smoke exposure tracking is, very roughly, (i) the estimation of smoke discharge from known characteristics of the fire, followed by (ii) predicting the spatiotemporal dispersion of the smoke using computational models including the effects of weather, topography, and so on. Sakiyama [22] and Yao et al. [30] describe one such system that is in use in Canada.

While existing air quality monitoring stations may provide some relevant data on exposure, their relevance is limited, because the location of the smoke event is not known in advance, and typically much of the smoke dispersion occurs over uninhabited regions where no stations exist. Hence, it is potentially quite beneficial to have even a presence/absence indication of smoke over the ROI. Such data could be used to inform the smoke dispersion models, or could be used on their own in studies of the health impacts of smoke.

### Previous Work

The literature on image segmentation is vast, as is the collection of machine learning and computer vision techniques with potential applicability to remote sensing data. That said, the amount of work on applying machine learning specifically to hyperspectral imagery is comparatively small. We recommend [21] as a modern overview of the field.

An initial study of smoke identification over the same ROI and time period as our dataset was reported by Wan et al. [25]. This study was exploratory in nature and considered only RGB colour images of the scene. It considered different approaches to pixel clustering based on the distribution of intensity in the red, green, and blue channels. While the results of the study showed promise, it was limited in a few respects. The colour channels were not modelled jointly, and all pixels were considered independent of one another. The study considered only clustering (unsupervised learning), so the accuracy of smoke predictions could not be quantified against labelled test images. Furthermore, the discriminatory power of the methods was inherently limited by the use of the colour images only, instead of the full set of hyperspectral data.

The work of Wolters and Dean [27] addressed some of these limitations by (i) using the hyperspectral images, (ii) modelling all of the spectral channels jointly through a logistic regression framework, and (iii) hand-labelling all pixels in the dataset as smoke or nonsmoke. The existence of pixel labels meant a supervised learning approach could be used. The available images were divided into training set (which was used to estimate model parameters), a validation set (used to do variable selection), and a test set (used to evaluate final model performance). It was found that while smoke plumes and other areas of thick smoke could be identified well, areas of low smoke concentration and areas with both smoke and cloud were difficult to predict correctly. This was attributed partly to labelling errors in the dataset, and partly to the inherent difficulty of separating the two classes.

The present work represents a further refinement of the methods of the previous two studies. We have considered improvements to the structure of our logistic regression model, and (most importantly from a technical standpoint) have incorporated spatial association among adjacent pixels by moving to an autologistic regression model. This model is described in the next subsection.

It is worth emphasizing that while the smoke identification problem has motivated our work, we view the autologistic regression classification procedure, outlined in Sect. 3, as the main contribution of this work. It is a general process that could be used to build automated image segmentation tools from any set of hyperspectral images with labelled training samples.

### Autologistic Regression

The autologistic model is a model for the probability distribution of a set of binary random variables with dependence described by a graphical structure. That is, the set of *n* variables may be considered vertices in an undirected graph; the edges in the graph encode the dependence structure among the variables. Its origins in the spatial statistics literature lie in the work of [3, 4], though it is equivalent to the much older Ising model of statistical physics. Models on undirected graphs are unified in the theory of Markov random fields (MRFs), which are common in model-based image processing methods (see e.g. [5]).

Autologistic *regression* models arise by incorporating covariate effects into the autologistic model. This allows the distribution of a set of binary random variables to be influenced by both covariate effects and the effect of neighbour variables through the graphical structure. Such models have been used in the ecological literature for studying regression problems related to the spatial characteristics of plant or animal species [1, 11, 12, 29].

Having assumed a graphical model for the binary responses, the entire set of variables (in our case, all the pixels in an image) must be modelled jointly. Let all of the pixels in an image be numbered from 1 to *n*, with binary random variable $Z_i$ representing the class of the *i*th pixel. For the moment, let the coding of the binary variables be unspecified, so that $Z_i$ can take values in $\{L,H\}$ (for “low” and “high”). Let $\mathbf {Z}$ be the random vector representing the classes of all pixels in an image. Then the joint probability mass function (PMF) of $\mathbf {Z}$ under the autologistic regression model is

1 $$\begin{aligned} \Pr (\mathbf {Z}=\mathbf {z})\propto \exp \left( (\mathbf {X}\varvec{\beta })^T\mathbf {z}+ \frac{\lambda }{2}\mathbf {z}^T\mathbf {A}\mathbf {z}\right) . \end{aligned}$$

Two terms appear in the exponent on the right-hand side. The first and second terms are referred to, respectively, as the *unary* and *pairwise* terms.

The unary term depends linearly on the binary responses $\mathbf {z}$. It is in this term that the regression component is incorporated. The vector $\mathbf {X}\varvec{\beta }$ is the linear predictor, where $\mathbf {X}$ is an $n\times r$ matrix of predictors derived from the hyperspectral image data; the *i*th row of $\mathbf {X}$ comprises the predictors for $Z_i$. The *r*-vector $\varvec{\beta }$ holds the corresponding regression coefficients.

The pairwise term is the part of the model that handles the spatial association. It is a quadratic form in $\mathbf {z}$, where $\mathbf {A}$ is an adjacency matrix of the graph. The coefficient $\lambda >0$ controls the strength of spatial association.

The model can also be expressed in a conditional logit form that aids interpretation. Letting $\pi _i$ be the conditional probability that $Z_i=H$, given all other variables in the graph, it can be shown that, for each *i*,

2 $$\begin{aligned} \log \left( \frac{\pi _i}{1-\pi _i}\right) = (H-L)\left( \mathbf {x}_i^T{\varvec{\beta }} + \lambda \sum _{j\sim i}z_j\right) , \end{aligned}$$

where the notation $j\sim i$ means that pixels *j* and *i* are neighbours according to the graph. This version of the model shows that the conditional logit of $\pi _i$ depends on the regression part plus an extra term: the coefficient $\lambda$ times the sum of pixel *i*’s neighbour classes. It is clear that if $\lambda = 0$, we have independence among the pixels and the model reverts to a standard logistic regression.

Throughout this work, we use a simple neighbourhood structure: our graph is a regular square grid, with each pixel connected to its immediate neighbours to the top, bottom, left, and right. Therefore, the sum in (2) has four terms whenever *i* is an interior pixel (and 2 or 3 terms if it is at the edge of the image).

### Estimation, Coding, and Centring

Despite the apparent simplicity of the conditional logit form (2), it should be remembered that the variables are in fact modelled jointly and that the coefficients $(\varvec{\beta }, \lambda )$ should properly be estimated simultaneously from the joint PMF (1). This represents the major technical challenge with the autologistic regression approach, because the PMF is specified only up to proportionality, and the proportionality constant is intractable (consisting of $2^n$ terms). Thus numerical optimization of the likelihood, for example, is impractical.

The most common (and simplest) alternative is maximum pseudolikelihood (MPL), which replaces the true likelihood for an image by the product of the conditional probabilities $\pi _i$ used in the logit form. Other approaches to the estimation of the autologistic model have also been considered. Hughes et al. [13] provide a good overview of the major options (as applied to the centred model described below). They compare MPL with Monte Carlo maximum likelihood and a Bayesian estimation approach. The most presently relevant of their conclusions were (i) that MPL is to be preferred for computational feasibility with large datasets, and (ii) that a high degree of spatial dependence (large $\lambda$) can make reliable inference hard for any of the methods.

Thus far, we have left the coding of the variables $Z_i$ unspecified. It is habitual in statistics to code binary variables as $\{0,1\}$, but for this model the zero/one coding leads to an undesirable asymmetry. Consider the sum $\sum _{j\sim i}z_j$ in (2). When pixel *i* has four neighbour pixels, each of which may take value zero or one, this sum can only take values 0, 1, 2, 3, or 4. Because the sum can never be negative, a pixel’s neighbours can never cause the log-odds that $Z_i=1$ to decrease, even when all four neighbours are 0 (the “low” value). The consequence is that estimates of $\varvec{\beta }$ and $\lambda$ are strongly coupled, and interpretation of the regression parameters becomes more difficult.

This asymmetry problem with the standard autologistic model has been noted and studied by Caragea and Kaiser [6] and Hughes et al. [13]. They concluded that a *centred* autologistic model is to be preferred. In logit form, the centred model is

3 $$\begin{aligned} \log \left( \frac{\pi _i}{1-\pi _i}\right) = \mathbf {x}_i^T{\varvec{\beta }} + \lambda \sum _{j\sim i}(z_j-\mu _j), \end{aligned}$$

where $\mu _j$ is the independence expectation of $Z_j$, that is, the expected value of the *j*th variable assuming $\lambda =0$.

It should be noted that the centred autologistic model was developed with the zero/one coding implicitly assumed throughout. In the present work, we take a different approach: we directly address the asymmetry by changing the coding to $Z_i\in \{-1,1\}$ without using the centring adjustment. It is easily seen that by letting *L* and *H* be symmetric around zero, the neighbour contribution $\sum _{j\sim i}z_j$ in (2) also becomes symmetric: the conditional log-odds of $Z_i=1$ will decrease if a majority of neighbours are negative, increase if a majority are positive, and be unchanged in the case of a tie. Aside from being more simple than the centred model, this approach enables a parameter estimation shortcut (described in the next section) that yields good predictive models with reasonable computational effort.

## Methods

Our goal is to build a classifier using supervised learning techniques with an autologistic regression model, given a collection of labelled hyperspectral images. It is assumed that there are from hundreds of thousands to millions of pixels per image, and that the number of images is sufficient for the problem to be considered data rich. By this, we mean that the number of pixels is more than sufficient for accurate statistical estimation; rather, the challenge is in using the data in a computationally efficient manner.

### An Estimation Shortcut for Large-Scale Classifier Construction

**Fig. 3:**
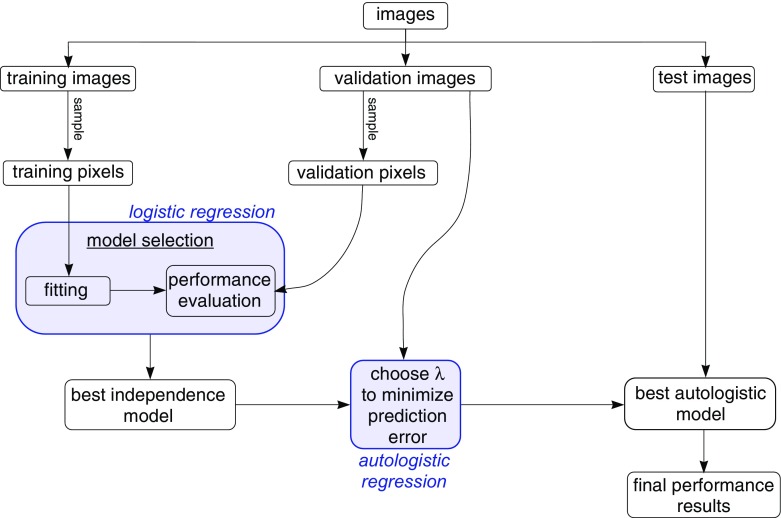
Analysis flowchart for classifier construction

Our proposal for analysis is shown pictorially in Fig. 3, which illustrates the flow of the data through the analysis procedure. Starting at the top, we see that the data are first split into training, validation, and test groups at the image level. Conventionally, the split of images among these groups might be $50/25/25\%$ [10]. The autologistic classifier is then built up in two stages, shown as shaded blocks in the figure. This two-stage approach is a distinctive feature of our proposal.

In the first stage, independence is assumed among pixels, and we endeavour to construct the best possible classifier using logistic regression. To do this, a large set of potential predictors (features) are first derived from the original hyperspectral data, and then an optimal subset of them is chosen. This involves repeated parameter estimation and performance evaluation. To do this in reasonable time, the fitting and model selection is not done using the full set of training and validation images; rather, large samples of pixels are taken from each of the groups.

The second stage takes the best logistic model and converts it to an autologistic regression by “plugging in” its parameter vector $\varvec{\beta }$, setting $\lambda$ to a nonzero value. The value of $\lambda$ is chosen to maximize predictive performance on the set of validation images. At this point, the classifier construction is finished and the final out-of-sample prediction performance can be evaluated using the test images.

Some results justifying the proposed plug-in approach and contrasting it with MPL estimation are given in Sect. 4, but a few comments on the proposal can be made here. First, the method depends on using the $\{-1,1\}$ coding for $\mathbf {Z}$. The symmetry introduced by this coding change appears to decouple $\varvec{\beta }$ and $\lambda$ sufficiently that the two parameters can be estimated separately and still produce a useful predictive model. Second, this approach allows the autologistic model to be viewed simply as a spatially smoothed logistic regression, with $\lambda$ acting as a smoothing parameter. As in other machine learning settings, we choose to set the value of this extra parameter directly based on a measure of predictive accuracy. Here, we are making use of the fact that our problem is purely predictive in nature. We are not interested, for example, in standard errors for the regression coefficients.^1^ Finally, estimating the parameters this way is prudent in our big data setting, where computational efficiency is a major concern. Performing model selection and estimation of $\varvec{\beta }$ with standard logistic regression is already computationally challenging, but it represents a major time saving over performing these steps with the full autologistic model.

The following two subsections fill in some details about the model selection and prediction steps of the process.

### Model Selection

The predictors $\mathbf {X}$ in (1) are not necessarily the hyperspectral data themselves; they may be any quantities derived from the original images. In building a classifier, it is helpful to start with a large collection of candidate predictors (features) and to select an optimal subset of these. In [27], for example, over 4000 candidate predictors were constructed from the hyperspectral data, their squares, square roots, and various interaction. In Sect. 5, bases for piecewise linear functions of the hyperspectral data are used.

In general, let $\mathbf {X}^*$ be a matrix containing all of the candidate predictors, one per column. We seek the subset of columns of $\mathbf {X}^*$ that provides optimal out-of-sample predictions. It may be expected that the number of columns in $\mathbf {X}^*$ is in the hundreds or thousands, while the optimal subset is comparatively much smaller. An exhaustive search through the possible subsets is impractical. Two reasonable approaches for model selection in this setting are shrinkage methods, and best-subsets search using heuristic methods. We take the LASSO [24] as prototypical for the former approach, and genetic algorithms (e.g. [16]) for the latter. We have used both of these methods and found they yield models with similar predictive capability. Our computing was done in the R environment [19], where packages glmnet [7] and kofnGA [26] can be used for shrinkage estimation and genetic algorithm search, respectively.

Because both the feature space and the amount of data are large, model selection is potentially time consuming even under the assumption of pixel independence. Even fitting a single logistic regression on the full dataset (which could consist of scores of megapixel images) could require advanced techniques due to local computer memory limitations.

The sampling step shown in Fig. 3 is proposed to reduce the computational burden of model selection. The sample size can be chosen so that parameter estimates and, more importantly, estimates of predicted probabilities, can be obtained with adequate accuracy in reasonable time. The best sample size to use will depend on the particular problem and the nature of the computational resources available, but preliminary trials should be sufficient to identify a reasonable choice. In our previous work with the smoke data, for example, we found that it was sufficient to sample $10^5$ pixels each for the training and validation sets. With this sample size, prediction error rates could be estimated to within the nearest percentage point.

### Prediction and Performance Evaluation

At both stages of estimation, the validation set is used to evaluate classifier performance using the fitted (predicted) probabilities, $\{\hat{p_i}\}$. The quantity $\hat{p_i}$ is the marginal probability that pixel *i* takes value $+1$ (smoke), under the fitted model. The fitted probabilities are used differently at each estimation stage.

At the model selection stage, where the logistic model is used under the independence assumption, we prefer to use the validation set deviance ($-2$ times the log-likelihood) as a measure of model quality. For any particular candidate model, this requires estimating the regression coefficients from the training pixels and then generating fitted probabilities for the validation pixels. These fitted probabilities are used to obtain the deviance. Deviance has been recommended elsewhere for model selection in logistic regression, particularly when using a shrinkage estimation approach [7].

In this first stage, both parameter estimation and performance evaluation are done using samples of the pixels in the training and validation images. One should be aware of the connection between pixel sampling and misclassification costs in our analysis scheme. The smoke data, for example, consist of about 90% nonsmoke and only 10% smoke pixels. If, in this case, we use simple random sampling to draw pixels for fitting the logistic regression model, we are implicitly prioritizing correct classification of nonsmoke over correct handling of smoke. Any incremental change in the classification error rate for the nonsmoke class will cost nine times as much (in terms of total error rate) as an equivalent change on the smoke class. One way to rectify this is to control the proportion of each class in the sampled pixels. In the analyses of the next two sections, for example, we require that each class makes up 50% of the final sample.

In the second stage where the autologistic model is used, a different approach is required. Pixels cannot be treated independently, so we do not sample pixels, and predictions are generated for whole images at once. A direct measure of prediction accuracy is then used, rather than a deviance. Given a set of estimated coefficients $(\hat{\varvec{\beta }},\hat{\lambda })$ and the image data $\mathbf {X}$ for a validation image, the approach is to first generate the fitted probabilities $\{\hat{p_i}\}$, and then compare these to a cutoff *c*. Any pixel *i* for which $\hat{p_i}>c$ is assigned to class 1; the rest are assigned to the $-1$ class. It is natural to set $c=0.5$, so that each pixel is assigned to the class with higher fitted probability, but it is possible that a different cutoff value is optimal, and one could search for the best choice of *c* in a given application. Previous work on the smoke data [27] explored different *c* choices and found that $c=0.5$ was appropriate, so we continue to use that value here.

The predicted classes can be compared to the actual classes of the validation images to produce a confusion matrix that tabulates the proportion of pixels of each class that were correctly and incorrectly identified. From this matrix it is possible to compute a variety of overall measures of performance: the overall error rate, the average error rate of the two classes, the Rand measure, the F-measure, and others. If we allow the cutoff *c* to vary, we could also consider integrated measures of quality like AUC (area under the receiver operating characteristic curve). The reader is referred to [18], and references therein, for more information.

There is no single best measure of classifier performance; the appropriate choice will depend on the application and, in particular, on the relative cost of the two types of error (e.g. the costs of predicting smoke when it is not there, versus the cost of missing a true smoke event). As in any quantitative evaluation, the measure used to define success should be chosen with care, since it will have a significant influence on the results. In the analyses to follow, we judge the two types of error to have equal weight, and simply use the total error rate as our performance measure.

A complication with the above procedure is that the probabilities $\{\hat{p_i}\}$ are not easily calculated for the autologistic model, owing to the intractable normalizing constant in the PMF. This problem is overcome by repeated simulation of realizations from the fitted model, which are then averaged to estimate the fitted probabilities. A few methods for simulating draws from an autologistic model are possible [2, 13, 23], but for our purposes, we use a Gibbs sampling approach [which is easy to implement because of the simple form of the full conditionals (2)].^2^

The final autologistic model with best parameter estimates $(\hat{\varvec{\beta }},\hat{\lambda })$ may subsequently be used to generate predictions on *new* images, where true class labels may or may not be known. This is done in the same way just described: the new image provides the $\mathbf {X}$ data for the fitted autologistic model, which is used to generate predicted probabilities by repeated simulation. The estimated probabilities are converted into binary predictions by comparison with the cutoff *c*.

## Analysis of Simulated Images

To test the validity of the approach just described, the methods have been applied to a set of synthetic images. Using simulated images has a number of advantages. There is no uncertainty about the true classes of the pixels; the number of images, their size, and the proportion of each class can be controlled; and the overall difficulty of the classification task can be controlled as well.

The primary goal with the simulated images was to investigate the validity of the two-stage estimation approach described in Sect. 3.1. To that end, the simulated images had only three planes. This allows them to be easily viewed as RGB images. It also eliminates the need for the model selection step, making it possible to focus on the performance of the estimation shortcut and the autologistic model.

### Generating the Images

The autologistic model is a conditional random field (CRF) image model [14]. Rather including a full generative model for the image data $\mathbf {X}$, the model takes $\mathbf {X}$ to be fixed and given. This is an advantage of the model—the daunting task of realistically modelling the image’s generating process is avoided—but it comes with the consequence that we cannot simulate random images from our model for test purposes. A different approach is needed.

The simulated images are to have two underlying true classes. One class may be thought of as the background (nonsmoke), and the other as connected regions of pixels overlaid on this background (smoke). The colours of each pixel are drawn from a distribution that depends on the true class, and also exhibits spatial correlation among pixels with the same class labels. To generate RGB images with these characteristics, the following procedure was followed:

1. Let an *n*-by-*n* image be mapped onto the unit square.
2. Generate a specified number of ellipses in the unit square with random foci and random major axis length. Pixels residing inside the union of these ellipses are assigned to the foreground (“smoke”) class; the remainder are assigned to the background class.
3. Generate realizations of three independent Gaussian Markov random fields (GMRFs) defined on the pixel lattice with $3\times 3$ neighbourhoods: one for red, one for blue, and one for green. Each GMRF is equal to a constant plus a zero-centred GMRF with user-specified parameters.
4. Use the antilogit function to transform the realizations from the previous step onto the [0, 1] scale.
5. Let the pixels in the background class take their R, G, B values from the corresponding pixels in the three GMRF draws obtained from steps 3 and 4.
6. Repeat steps 3 and 4 with alternative parameters. Let the pixels in the foreground class take their R, G, B values from the corresponding pixels in these new GMRF draws.

Using random ellipses allows us to obtain “smoke” regions with random shapes, and the total amount of smoke also varies from image to image. The shape characteristics and proportion of smoke can be modified by varying the parameters controlling ellipse generation. For the present study, we used 25 ellipses per image.

**Fig. 4:**
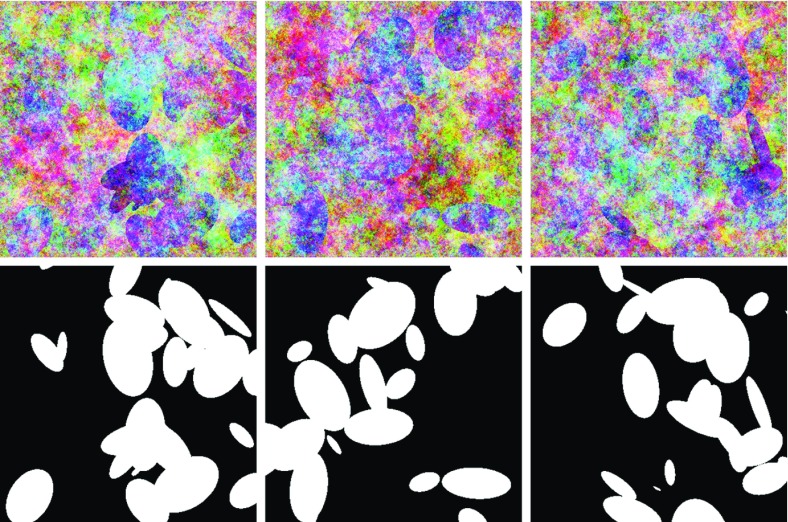
Examples of the simulated images. *Top row* the images. *Bottom row* the true classes

Varying the parameters of the GMRFs allows control of the within-class spatial smoothness as well as the between-class colour differences. In generating the GMRFs, the constant vector (R, G, B) was set to (on the [0,1] scale) (0.75, 0.65, 0.55) for the background and (0.6, 0.5, 0.7) for the foreground. These parameter settings were chosen to make the classification task a challenging one, not unlike the smoke identification task. At these settings, the foreground class is generally more blue/purple and the background class is generally more red/yellow, but there is considerable noise and significant overlap between the two classes. Figure 4 provides three example images with their true classes. Even a human being with knowledge of the elliptical nature of the foreground class would have difficulty achieving perfect classification.

Images were generated at five different sizes: 100, 200, 400, 600, and 800 pixels square. Ninety images were generated for each size, and these were divided equally into three groups for training, validation, and testing.

**Table 1:** Results of MPL and plug-in estimation for different image sizes

Pixels	Method	$\hat{R}$	$\hat{G}$	$\hat{B}$	$\hat{\lambda }$	Error rate (%)	Time (min)$^\mathrm{a}$
$100^2$	plug-in	$-2.21$	$-2.02$	1.91	0.90	20.1	0.25
	PL	$-2.04$	$-1.99$	2.06	0.99	20.4	0.49
$200^2$	plug-in	$-1.64$	$-1.35$	1.71	1.00	17.7	0.66
	PL	$-1.61$	$-1.30$	1.70	1.19	17.7	1.5
$400^2$	plug-in	$-2.05$	$-1.42$	1.63	1.60	20.1	2.8
	PL	$-2.08$	$-1.40$	1.68	1.36	20.1	7.5
$600^2$	plug-in	$-1.91$	$-1.22$	1.76	1.95	20.6	6.9
	PL	$-1.97$	$-1.36$	1.79	1.51	20.4	20
$800^2$	plug-in	$-1.55$	$-1.44$	1.58	1.95	18.8	12
	PL	$-1.57$	$-1.43$	1.49	1.59	18.6	35

Symbols $\hat{R},\hat{G},\hat{B}$ denote coefficients for the red, green, and blue predictors, respectively. These coefficient values correspond to the plus/minus response coding. The two estimation methods consistently give similar parameter estimates and error rates

$^\mathrm{a}$ Reported times for the plug-in method are times for a single $\lambda$ value in a parallel computing implementation; see the comments in Sect. 6

### Predictive Performance

Each image size was analysed separately, using the procedure of Fig. 3 (but omitting the model selection step). The independent-pixel logistic model was fit using a sample of $10^5$ pixels from the training images, with 50% of the sample having each class. The predictors for the logistic model were the red, green, and blue colour intensities in the image. The pairwise parameter in the autologistic model was estimated by the plug-in approach: predictions were generated for candidate values $\lambda =0,0.05,0.1,0.15,\ldots ,2$, and $\hat{\lambda }$ was set to the value that minimized the validation set deviance.

For comparison purposes, the model parameters were also estimated by maximum pseudolikelihood. The best plug-in model and the MPL model were then used for performance evaluation, as measured by test-set prediction error rate.

Table 1 summarizes the results. It shows the regression coefficient and pairwise coefficient estimates, along with the overall error rate and run time, for each method at each image size.

Considering first the coefficient estimates, it is seen that the two estimation methods produce remarkably similar regression coefficients for all image sizes. This is despite the fact that MPL estimates $(\hat{\varvec{\beta }},\hat{\lambda })$ simultaneously and the plug-in method does so in two stages. The pairwise coefficients also show reasonable agreement, though the differences are larger than for the regression parameters. All of the estimated pairwise parameters are quite large, indicating a high degree of spatial smoothness in the fitted models.

**Fig. 5:**
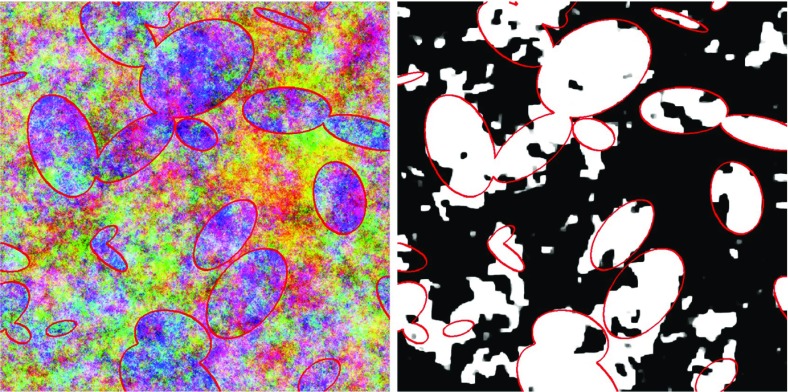
Example of prediction results for an $800\times 800$ test image. *Left* the original image. *Right* the predicted probabilities using plug-in estimation. True class boundaries have been superimposed to aid visualization

The similarity of the parameter estimates suggests that the two fitted models should have similar predictive abilities, and the error rates confirm that this is the case. Error percentages vary from about 18 to about 20, depending on the image size. Figure 5 gives an example $800\times 800$ image from the test set, along with the predicted probability map produced by the plug-in model. The autologistic model has caused the map to be free of short-range noise. Qualitatively speaking, the predictions display a good agreement with the visual appearance of the original image. Locations where predictions were incorrect appear to correspond to areas where the colour distributions of the two classes truly overlap.

The table also lists run time measurements for each case. Comparison of MPL with the plug-in approach is not straightforward, however, because implementation details can have a large impact on run time. A discussion of computational aspects is deferred to Sect. 6.

### Effect of Coding and Centring

The preceding results showed that the two-stage estimation procedure gave reasonable parameter estimates and predictions. Increasing $\lambda$ from 0 while keeping the unary parameters fixed caused improvements in prediction, due to reduced noise in the predicted probabilities. In Sect. 2.4, it was claimed that this effect is a consequence of using the $\{-1,1\}$ variable coding. Here we provide evidence to support this claim.

To compare different variants of the autologistic model, the plug-in estimation procedure was repeated on the $400\times 400$ simulated images using the standard model (2) with $\{0,1\}$ coding, as well as the centred model (3) with both $\{0,1\}$ and $\{-1,1\}$ coding. The overall prediction error rate on the test set was recorded for various $\lambda$ values for each model variant.

**Fig. 6:**
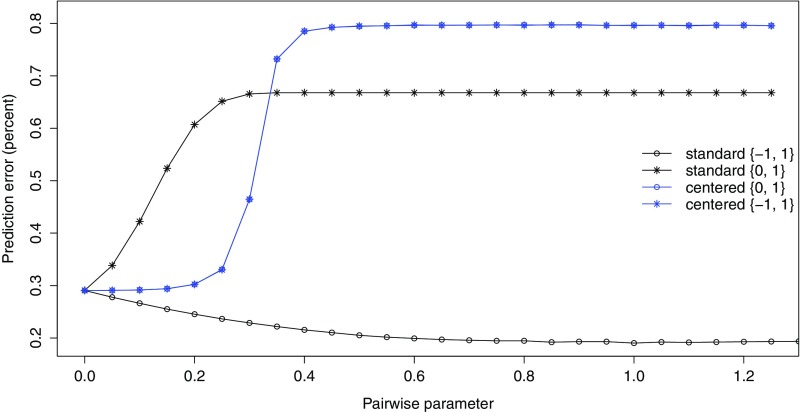
Effect of plugging in various $\lambda$ values on prediction error, for different autologistic model variants. Only the standard model with $\{-1,1\}$ coding shows improvement; the other models show a strong degradation of performance. Pairwise parameter values for all variants are converted to the $\{-1,1\}$-coding scale

The results are shown in Fig. 6. At $\lambda =0$, all variants give the same performance, since all of them are equivalent to the independence model. As $\lambda$ increases, however, only the standard $\{-1,1\}$ model shows an improvement. The standard $\{0,1\}$ model immediately begins to show a decline in performance. The two centred models give results so similar that their curves are indistinguishable from each other: they first remain nearly constant, only to show a rapid increase in error rate above about $\lambda =0.2$. This demonstrates that the plug-in estimation approach and its computational benefits are *only* viable with the standard $\{-1,1\}$ model. For all of the other variants, simultaneous estimation of $(\varvec{\beta },\lambda )$ would be required to obtain any improvements over logistic regression. This result is somewhat surprising, given the seemingly minor differences among the models. A few additional comments on this topic are given in Sect. 6.

## Application to the Smoke Data

The methodology of Sect. 3 was also applied to the smoke identification dataset described in Sect. 2.1. As this is a real-world hyperspectral dataset, the complete procedure illustrated in Fig. 3 was carried out. Below, we first outline how model selection and parameter estimation were done, and then give prediction results from the best model found.

### Model Selection and Plug-In Estimation

Model selection and regression parameter estimation were done using training and validation samples of $10^5$ pixels. Each sample was restricted to consist of 50% smoke pixels and 50% nonsmoke pixels.^3^

The 35 observed predictors were hyperspectral bands 1–28 and 30–36 (band 29 was not available due to a hardware problem on the instrument). We can refer to these predictors as main effects.

Two classes of models were considered: first, those models consisting of only main effect terms; and second, those models consisting of main effects or two-way interactions among the main effects. In each case, models of size $3,4,6,8,10,\ldots ,18$ were considered. Model search was carried out using a genetic algorithm, with validation set deviance as the objective to be minimized.

To improve the flexibility of the models, a generalized additive model [GAM; 15 was used, with each factor’s effect on the log-odds modelled as a piecewise linear function. Say, for example, that our model search is presently considering a model with band 2, band 3, and the interaction of bands 4 and 5 as predictors. In this case the independent-pixel model is

$$\begin{aligned} \log \left( \frac{\pi _i}{1-\pi _i}\right) = \beta _0 + f_2(x_{i2}) + f_3(x_{i3}) + f_{4:5}(x_{i4}x_{i5}), \end{aligned}$$

where $x_{ij}$ represents the image value for pixel *i* in band *j*, and the functions $f_\cdot ()$ are piecewise linear on [0, 1]. Six triangular basis functions were used for each piecewise function, resulting in five coefficients to be estimated for each factorial effect.^4^

**Table 2:** Information about the best models found

Predictor set	Selected variables (MODIS band numbers)	plug-in $\hat{\lambda }$
Main effects	1 6 7 8 14 16 17 18 21 23 25 26 30 31 32 36	1.85
Main effects & interactions	7 30 2:3 5:26 6:11 7:36 8:20 8:22 8:25 8:31 13:15 13:23 16:31 18:23 22:36 32:36	1.75

It happened that the overall minimum-deviance model for both the main-effects only and the interactions-included models had 16 predictors. For reference, the selected variables in each best model are given in Table 2. Inspection of the estimated piecewise functions (not shown) showed many nonlinearities and some sign changes. Meaningful interpretation of these models is hard, however, due to the large number of variables included.

The table also shows the values of $\hat{\lambda }$ found by plug-in estimation. For both models, a large value was chosen, indicating a high degree of spatial smoothing.

Of the two best models shown in the table, the interactions model had lower deviance (as would be expected, given the larger pool of models it was drawn from). Hence, the best 16-variable interactions model was taken as the final selection moving forward, and the plug-in value $\hat{\lambda }=1.75$ was used to make it an autologistic model for final performance evaluation on the test images.

### Prediction

Table 3 shows the test-set error rates for the best main effects model and the best interactions model, both with $\lambda =0$ (logistic model) and with $\lambda =\hat{\lambda }$ (autologistic model). It is clear that both adding in the interaction terms and including the pairwise association have a beneficial impact on predictive ability.

The table shows both the class-conditional error rates as well as the overall rate. In this case, we used the overall error rate as our objective, and trained our classifiers on samples with equal numbers of smoke and nonsmoke pixels—essentially, we sought to minimize overall error rate while keeping the class-conditional rates reasonably balanced. It appears from the table that we achieved this result. We view the best model’s overall error rate of 16.7% as quite successful, given the challenges of this dataset (which are touched on in Sect. 6.3).

**Table 3:** Test-set prediction error rates for the smoke data

	Error rate (%)		
Model	Nonsmoke pixels	Smoke pixels	Overall
Main effects, logistic	21.1	25.9	21.6
Interactions, logistic	20.0	23.3	20.3
Main effects, autologistic	17.6	23.9	18.2
Interactions, autologistic	16.2	21.3	16.7

Adding interaction terms and using the autologistic model both yield improvements over the base logistic regression model

**Fig. 7:**
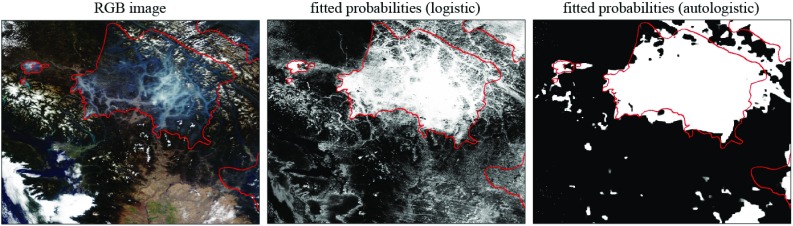
A scene that is relatively easy to segment, showing the colour image and the fitted probability maps for the logistic and autologistic models. *Red outlines* indicate the boundaries of the human-identified smoke regions

**Fig. 8:**
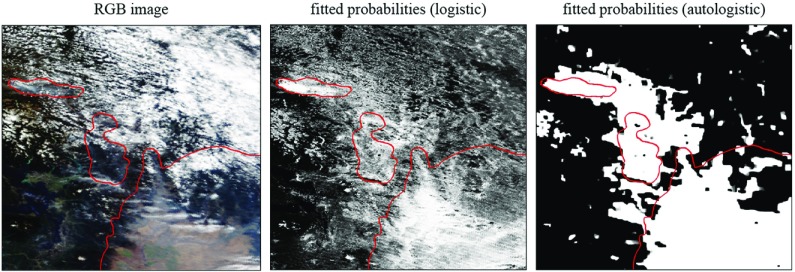
A scene that is harder to segment due to the presence of clouds and areas with thin smoke. The autologistic model imparts spatial smoothness but cannot correct ambiguities inherent in the logistic model

Figures 7 and 8 provide visual examples of the best model’s predictions. Each figure shows only part of the ROI. Figure 7 gives an example of an image that is relatively easy to segment. The RGB image on the left shows that the sky is largely cloud-free, and the smoke in the image is thick (recall that this image is only used to aid visualization; classification is based on the full hyperspectral data). The middle image shows the predicted probability map from the independent-pixel model, while the rightmost image shows the corresponding map using the autologistic model. The effect of including spatial association is very clear. In the largest smoke region, classification is very good. The image does include some areas of false-negative predictions (which are associated with “thin” smoke areas) as well as some false-positive regions (which are associated with regions of cloud or snow).

Figure 8 gives the RGB image and the autologistic prediction map for a scene that is more challenging to classify. Although the thick smoke areas are correctly identified, significant areas of both false-positive and -negative predictions are visible.

## Discussion

This section expands somewhat on three topics that have arisen in the preceding sections: the effect of coding and centring, the computational requirements of the method, and practical considerations that arose in the smoke data analysis.

### More on Coding and Centring

Figure 6 is one of the more interesting results in this work. It draws a sharp distinction between the standard autologistic model with $\{-1,1\}$ coding and the other autologistic variants. Two observations may help to understand the figure:

1. The plug-in estimation approach gives markedly different results for the two standard models with a different coding. This is a result of the previously mentioned asymmetry inherent in the $\{0,1\}$ coding. This asymmetry is alleviated by changing to the plus/minus coding.
2. In contrast, the centred model gives the same result regardless of coding; but this result involves no benefit. It seems that the centring adjustment eliminates the asymmetry problem, but in a way that does not allow us to interpret $\lambda$ as a smoothing parameter. Joint estimation of $(\varvec{\beta }, \lambda )$ would still be required to achieve good performance.

A deeper understanding of the differences among the model variants would involve a study of their equivalence and how they may be transformed into one another. Importantly, we note that changing the coding or centring in an autologistic regression is more than a parameter transformation; it actually results in a different probability model. This can explain why the centred models seemingly resolve the problem of asymmetry—the models give identical results with either coding—while still being unsuitable for plug-in estimation. We are currently studying the properties of the coding schemes and have found that the standard model with $\{-1,1\}$ coding has uniquely desirable properties among the autologistic variants; we expect to publish results on these properties in the near future.

It is also suggestive that the MPL and plug-in estimates of Table 1 are so similar. Theoretical work on the quality of plug-in estimates (and the conditions under which plug-in estimation is reasonable for prediction) is another potentially fruitful area for future investigation.

### Computational Considerations

The estimation run times in Table 1 were included to facilitate a few remarks about the computational aspects of estimation. The task of computing the MPL estimates is relatively straightforward. For estimation using a collection of *m* images, each *n*-by-*n*, the (log) pseudolikelihood is a sum of $mn^2$ terms. Any continuous optimization routine can be used to find estimates of $\varvec{\beta }$ and $\lambda$, but the objective function becomes slow to compute as *n* grows. This is seen in the table, where the time to obtain MPL estimates rose from about half a minute for $n=100$, to 35 min for $n=800$. One could consider parallelization to improve performance, but this sort of computation (a costly objective function involving a large number of small computations, inside an optimization routine) is not trivial to parallelize efficiently.

For the plug-in method, the computational considerations are different. Conventional optimization is only done for the independence model, where pre-existing routines can be used to get estimates efficiently even for large datasets. Instead, the computational overhead of the plug-in method is almost wholly due to the need for sampling to estimate the marginal probabilities $\{p_i\}$ to generate predictions. For a set of *m* images and *v* candidate values of $\lambda$ a total of *mv* sampling runs (in our case, Gibbs samplers) must be carried out. Although sampling is computationally intensive, parallelization of sampling across the *m* images is very easy, since the task is a set of lengthy, independent, computations. The plug-in run times in Table 1 show the time required for performance evaluation at a single candidate $\lambda$ value using 10 worker processes on a 6-core workstation with hyperthreading.

As a final remark about run times, recall that in a real application with hyperspectral data, model selection will be an important component of the estimation process. In the smoke identification application with GA search across many model sizes, the model selection stage took approximately two days with computations parallelized on the same six-core workstation. Because we used the two-stage estimation approach, model selection only involved fitting standard logistic regressions. If instead we had used MPL estimation, search time would take an impractical length of time.

### Lessons from the Smoke Data

We view the results on the smoke images as positive, despite the still relatively high error rates. This is for two reasons.

First, pixels were labelled as smoke even if the smoke was thin or indistinct, and even if there was also cloud present. This was done with awareness that it would make classification more difficult, in the spirit of testing the limits of the methodology. Much lower error rates may be expected if we instead focus on the easier target of identifying clear-sky areas with thick smoke or smoke plumes. Importantly, thick smoke is more important from a health standpoint, so the methods of this article remain of interest for smoke identification.

The second factor likely contributing to misclassification is a significant proportion of labelling errors in the training data. It was stated in the introduction that our methods are designed for cases where a human photointerpreter can identify the feature of interest, but the smoke data only partially fit that description. The “true” smoke regions were identified using only the RGB images, not the full hyperspectral data. When smoke is diffuse or is obscured by cloud, it is more difficult to correctly label all pixels.

A few options exist to ameliorate the aforementioned problems. For example, a multi-class labelling approach could be taken to handle both thick and thin smoke areas. Our methods could also be adapted to a semi-supervised learning setting, where only some of the image pixels are hand-labelled, or where clustering is employed to establish natural groups of pixels (as in [21], Sect. 11.4). The semi-supervised approach would also be attractive if trying to meld satellite data with ground truth measurements from a limited number of monitoring stations. The autologistic model would remain a means to introduce spatial association into such approaches.

Development of an independent-pixel logistic model with good predictive power is essential for the methods described here to be effective. Expanding the logistic model into an autologistic one can provide spatial smoothness, but will not be sufficient to repair a poorly performing logistic model. It is important in an application, then, to take care to build a good logistic model through appropriate data processing and feature selection. To that end, we view the GAM approach as a promising option for large-scale hyperspectral image classification problems. It allows nonlinear relationships between the predictors and the responses to be learned in a data-driven manner during training, without introducing excessive computational burdens during the model selection stage. Depending on the application and the size of the candidate model, it is also possible that the GAM component functions could provide useful scientific interpretations.

There are other potential additions and extensions of the autologistic regression model that were not considered explicitly here: spatiotemporal modelling, including other covariates, and background subtraction.

The spatial autologistic model could be extended to a spatiotemporal one, simply by extending the graph representation of the data. This would involve at least one additional parameter to handle the temporal effect. For the smoke data, we discarded the idea of including the temporal component early on, because the images exhibited huge variations in smoke and cloud cover from day to day. A finer time resolution would be required to derive benefit from spatiotemporal modelling.

Incorporating additional covariates, not originating in the raw images, into the analysis is another alternative that is easily implemented. Measures of ground cover or land use, for example, could be straightforwardly included by expanding the $\mathbf {X}$ matrix of predictors. Including these as categorical variables in the model and allowing interactions would make the regression model more flexible to adapt to different ground-cover types.

Finally, background subtraction is a technique to consider to improve the quality of regression models built from image data. This involves using a set of background-subtracted images, where a median background image (the hyperspectral equivalent of the top image in Fig. 2, arising from smoke-free days with no forest fire events) would be subtracted from all images before further analysis. Development of a background image involves its own complications (image registration, time-varying background characteristics, land use changes, and so on), but where such an image can be established it is worth considering background subtraction. In our case, preliminary trials with the subtracted images showed no benefit in terms of prediction accuracy, so we used only the original unmodified images in the final analysis. Even so, further work in this direction is being considered.

## Conclusions

Remote sensing imagery is a potentially significant source of auxiliary data for quantification of human health hazards. The comprehensive spatial coverage of satellite-borne imaging products is particularly attractive, as it can augment ground-based observations from monitoring stations.

Identifying a well-defined hazard in a remote sensing image is a binary image segmentation problem. The autologistic regression model is attractive for such problems. It is a natural extension of logistic regression, allowing model-based spatial smoothing in the predicted segmentations. Standard methods of estimation and prediction, however, present a prohibitive computational burden when using this model with large collections of megapixel-scale hyperspectral images.

The method presented here, summarized in Fig. 3, makes the autologistic regression approach feasible for large data sets of this type. The key feature of the proposed approach is two-stage estimation of the regression coefficients and the pairwise association parameter. Estimating the regression parameters under the assumption of independence leads to huge computational savings. It allows estimation to be done based on a sample of the training pixels, using standard logistic regression code. Equally important, the improved computational efficiency makes it possible to consider a larger space of candidate features, that will lead to better classifier performance.

The two-stage estimation has been made possible by the simple device of coding the binary variables $\{-1,1\}$. The benefits of using this approach cannot be achieved using the more standard $\{0,1\}$ coding, or using the centred autologistic model.

It was shown with simulated images and a real smoke identification example that the proposed approach gives promising prediction results. The autologistic model behaves in the desired manner, that is, as a spatially smoothed logistic regression classifier. The methodology should be readily applicable to other hyperspectral image segmentation applications where labelled training samples are available.

## Footnotes

1 Obtaining standard errors for $\varvec{\beta }$ is challenging regardless of the estimation approach. We expect that resampling methods would enable us to obtain standard errors with the plug-in estimation procedure, and that these would be comparable to those obtained when $\varvec{\beta }$ and $\lambda$ are estimated jointly. As mentioned, this avenue was not pursued given the prediction-focused nature of the segmentation task.

2 We have found no drawbacks to using Gibbs sampling, and found that runs of approximately 400 draws were sufficient to get good estimates of the marginal probabilities. Furthermore, it is only necessary to obtain good estimates of whether $p_i>c$, not of the $p_i$s themselves.

3 In previous work [27], we used 90% nonsmoke pixels in the samples, to match the population proportion. This resulted in lower overall error rates than those reported here, but very high false-negative rates among the truly smoke pixels.

4 Each function was set to have $f_\cdot (0)=0$ for identifiability. The functions’ boundary knots were set to 0 and 1, with interior knots evenly spaced between the 10th and 90th percentiles of the training data values.
